# Mitochondrial Intercellular Transfer via Platelets After Physical Training Exerts Neuro‐Glial Protection Against Cerebral Ischemia

**DOI:** 10.1002/mco2.70590

**Published:** 2026-01-15

**Authors:** Toshiki Inaba, Nobukazu Miyamoto, Kenichiro Hira, Chikage Kijima, Yoshifumi Miyauchi, Hai‐Bin Xu, Kazo Kanazawa, Yuji Ueno, Nobutaka Hattori

**Affiliations:** ^1^ Department of Neurology Juntendo University School of Medicine Tokyo Japan; ^2^ Department of Neurology University of Yamanashi Yamanashi Japan; ^3^ Neurodegenerative Disorders Collaboration Laboratory RIKEN Center for Brain Science Saitama Japan

**Keywords:** cerebral infarction, mitochondrial migration, platelet therapy, prolonged cerebral hypoperfusion, white matter injury

## Abstract

While thrombolytic therapy can be effective for stroke, many patients are unable to benefit due to time restrictions. In an aging society, sarcopenia, a condition marked by reduced muscle volume, often worsens recovery after stroke. Our study explored how mitochondria, which are abundant in muscle, could aid in stroke recovery through exercise‐induced migration. Using mouse models of chronic hypoperfusion and ischemia, alongside in vitro studies with rat primary cells under oxygen–glucose deprivation and CoCl2 exposure, we found that treadmill exercise protected against white matter injury, myelin loss, astroglial formation, and memory deficits observed 28 days post‐hypoperfusion. In acute ischemia models, training reduced glial activation and post‐stroke complications. Exercise increased mitochondrial levels in muscle and blood, facilitating their migration between tissues via platelets. In vitro, the addition of muscle‐derived mitochondria enhanced the survival of neurons, astrocytes, and oligodendrocytes. Notably, platelets carrying mitochondria from treadmill‐trained mice significantly improved ischemic white matter injury and mitigated post‐stroke complications. This study highlights mitochondria as a critical part of the secretome, suggesting that muscle‐derived mitochondria might play a role in the protective effects of remote ischemic preconditioning. Cell–cell mitochondrial migration, therefore, could offer a promising new approach to reducing post‐stroke complications and vascular dementia.

## Graphical abstract

Treadmill training protects against white matter injury and reduces cerebral infarction.

Muscle‐derived mitochondrial transfer via platelets is promoted by treadmill training.

Platelet transfusion emerges as a potential low‐risk therapeutic strategy for sequelae and vascular dementia.

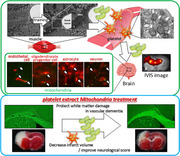

## 1 Introduction

The most effective strategy for achieving functional recovery in cerebral infarction involves a combination of thrombus retrieval and thrombolytic therapy [1]. Although the therapeutic time window has recently been extended, those treatments remains effective only within a narrow window of 6 h after onset. Beyond this period, management shifts to symptomatic treatment and strategies aimed at limiting infarct expansion. Japan and many other countries are predicted to have a super‐aged society; thus, dementia and sarcopenia/frailty threaten to reduce healthy life expectancy [2].

Dementia is broadly classified into Alzheimer's disease, characterized by neuronal degeneration in the hippocampus and cerebral cortex, and cerebrovascular dementia, which is associated with deep white matter lesions caused by cerebral microvascular disease. Emerging evidence indicates that microcirculatory disturbances and white matter injury also accelerate the progression of Alzheimer's disease, suggesting that cerebrovascular pathology and cortical neurodegeneration jointly contribute to cognitive decline [3]. White matter lesions are therefore thought to underlie a large proportion of dementia cases. Astrocytes and oligodendrocytes (OLGs), which support axons and myelin sheaths in white matter, play critical roles in maintaining neural integrity, yet the mechanisms underlying their vulnerability to ischemic and metabolic stress remain incompletely understood [4].

Sarcopenia and frailty, characterized by reduced muscle mass and physical activity, are highly prevalent in elderly stroke patients and are strongly associated with poor functional recovery [5]. Exercise‐based rehabilitation, including treadmill training, has been reported to improve post‐stroke outcomes, potentially through the induction of circulating cytokines such as myokines [6, 7]. However, the biological mechanisms underlying these beneficial effects remain poorly defined, and the clinical efficacy of exercise interventions varies widely among individuals. Skeletal muscle is a mitochondria‐rich organ, and reduced muscle mass has been associated with poor outcomes after myocardial infarction [8]. These observations raise the possibility that patients with sarcopenia and frailty may have diminished systemic mitochondrial availability, thereby limiting tissue repair and functional recovery after ischemic injury [9]. Furthermore, skeletal muscle mass is closely linked to mitochondrial function, suggesting that mitochondrial intercellular transfer may contribute to the pathophysiology of sarcopenia [10].

Recent studies have highlighted mitochondrial transfer as a novel form of intercellular communication involved in tissue protection and repair [11]. Initially recognized as a mechanism for metabolic support of injured cells, mitochondrial transfer is now understood to regulate mitochondrial quality control, tissue homeostasis, and tissue remodeling [11]. Mitochondria can be transferred through several pathways, including tunneling nanotubes, dendritic projections, gap junctions, and extracellular vesicles [11]. Importantly, circulating blood components—particularly platelets—have emerged as potential mediators of mitochondrial transfer. Platelets contain functional mitochondria and are rapidly activated by physiological stimuli such as exercise. Although mitochondria derived from endothelial progenitor cells and amniotic cells have shown neuroprotective effects in cerebral ischemia models, the contribution of platelet‐mediated mitochondrial transfer to brain ischemia and white matter injury remains largely unexplored [12, 13].

Cell transplantation therapy has demonstrated beneficial effects in chronic‐phase cerebral infarction [14], partly through neuronal replacement and bystander effects mediated by secreted factors [15]. However, high costs, time‐consuming preparation, and strict facility requirements limit its widespread clinical application [16]. In contrast, exercise‐induced endogenous mechanisms represent a potentially safe, scalable, and sustainable therapeutic strategy aligned with the goals of extending healthy life expectancy under the Basic Act on Stroke and Cardiovascular Measures [17, 18, 19]. Recent advances in rehabilitation, including robotic exoskeletons such as HAL [20], virtual reality‐based motor training, and creative therapies, have shown promise; however, early initiation of rehabilitation remains the most consistently effective factor for improving functional outcomes [21]. Despite these advances, more than half of stroke survivors continue to experience significant long‐term disability.

In the present study, we first examined whether exercise exerts protective effects against cerebral infarction and ischemic white matter injury, with a particular focus on mitochondrial cell–cell interaction. Based on our initial findings suggesting the possibility of platelet‐mediated intercellular mitochondrial transfer, we next investigated whether platelets obtained from exercise‐loaded animals—referred to as mitochondria‐rich platelets—could reproduce these protective effects when administered to recipient animals. Through this stepwise approach, we aimed to clarify the contribution of platelet‐derived mitochondrial dynamics to exercise‐induced neuroprotection.

## 2 Results

### 2.1 Training Affects Cognitive Function After Prolonged Cerebral Hypoperfusion

**FIGURE 1:**
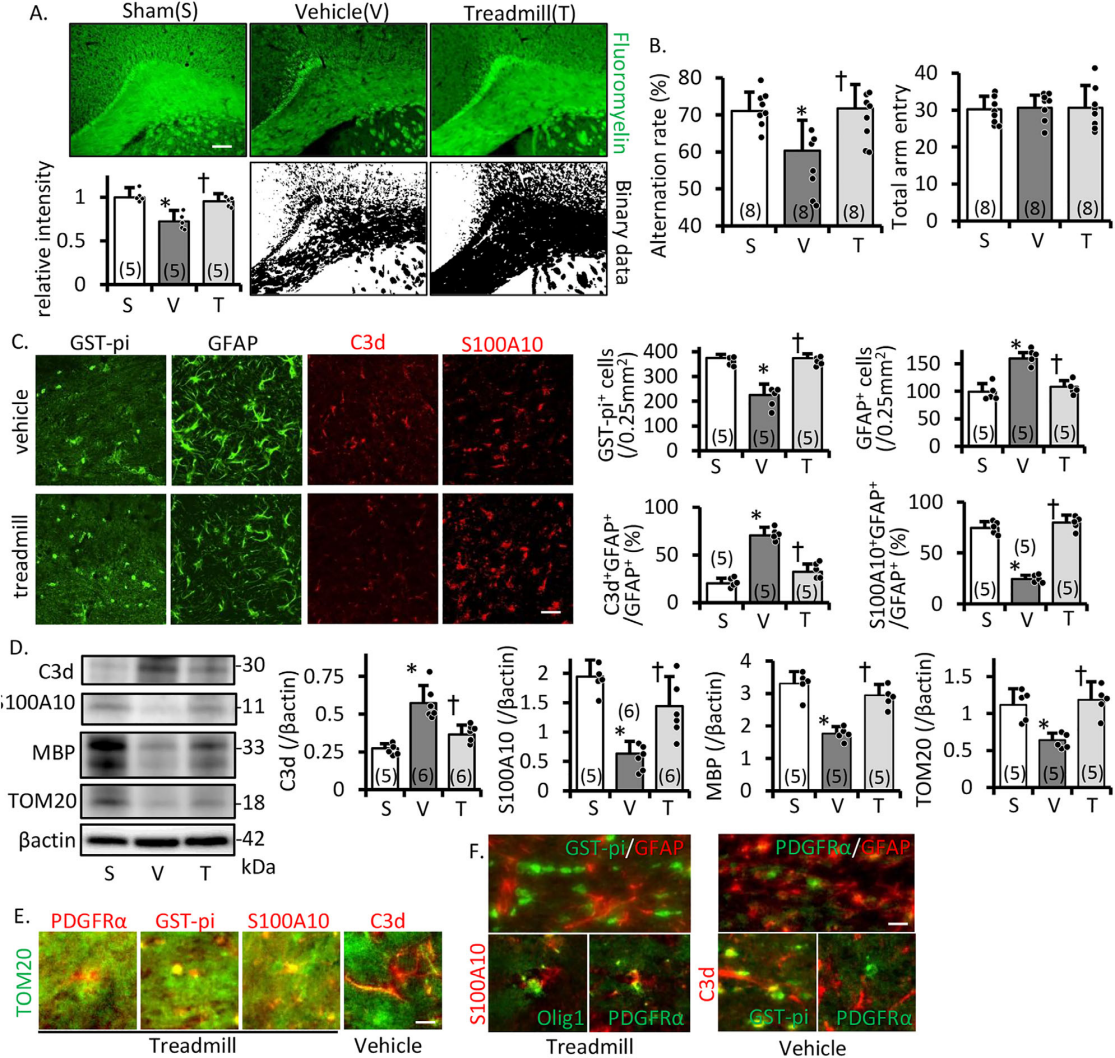
Prolonged ischemic white matter injury in the BCAS model of treadmill/vehicle group. Serial changes in astrocytes, and inflammatory/protective astrocyte typing in the corpus callosum. (A) FluoroMyelin staining and relative FluoroMyelin intensity in the corpus callosum at 28 days after the BCAS operation. Scale bar, 100 µm. (B) Spatial memory by the spontaneous Y‐maze test, alternation rate, and locomotor activity (total arm entry). Treadmill training protected against white matter damage progression and recent memory disturbance. (C) Representative image and intensity of GST‐pi (green), GFAP (green), C3d (red), and S100A10 (red) immunostaining of the cerebral corpus callosum on day 28 after BCAS. Scale bar, 50 µm. (D) Representative image and densitometric analysis of western blotting of the corpus callosum on day 28 after BCAS. Treadmill training protected astrogenesis and maintained oligodendrocyte number, myelin density, and protective astrocytes under hypoperfusion. (E) Representative images of PDGFRα (red), GST‐pi (red), C3d (red), S100A10 (red), and TOM20 (green) immunostaining of the cerebral corpus callosum on day 28 after BCAS in treadmill and vehicle groups. Scale bar, 25 µm. TOM20 merged with PDGFRα, GST‐pi, and S100A10, but not with C3d. (F) Representative images for PDGFR𝛼, GST‐pi, Olig‐1 (mature oligodendrocyte maker), C3d, and GFAP in the white matter of mice before, and day 28 after BCAS. Scale bar, 25 µm. S, sham; V, vehicle; T, treadmill group. Data are the mean ± SD of *n* = 5–6, except B; *n* = 8. *p ≤ 0.05 compared to the sham group, †*p* ≤ 0.05 compared to the vehicle group.

We analyzed the effect of treadmill training (three times/week, 8 m/min, 30 min) on cerebral white matter damage after cerebral hypoperfusion. At 28 days after BCAS operation, ischemic white matter damage progressed on myelin staining (Figure 1A). Spatial memory disturbance without locomotor disruption (arm entry) on Y‐maze test had progressed in the vehicle treatment group. In the treadmill training group, white matter injury and spatial memory dysfunction did not progress at 28 days after BCAS. Progression was similar to the sham group (Figure 1B). In the corpus callosum, GST‐pi‐positive cells (mature OLG maker) were decreased. GFAP (astrocyte maker) was increased in the vehicle group. With treadmill training, GST‐pi/GFAP‐positive cells were almost the same level as the sham group (Figure 1C). Western blotting (WB) revealed that MBP, a mature OLG maker, significantly increased in the treadmill group compared to the vehicle (Figure 1D).

We previously reported that C3d^+^ inflammatory astrocytes increased after BCAS operation and S100A10^+^ protective astrocytes increase mitochondrial projection and protect the white matter environment [12]. Therefore, we checked the ratio of inflammatory/protective astrocytes. Treadmill training decreased inflammatory astrocytes and increased protective astrocytes even in BCAS operation (Figure 1C,D). TOM20 band intensity (mitochondrial maker) in the corpus callosum was increased in the treadmill group compared to the vehicle (Figure 1D). TOM20 was merged with S100A10, PDGFRα (OLG progenitor maker), CD31 (micro vessel maker), and GST‐pi, but not C3d (Figure 1E). Moreover, OLG lineage cells were found to exist in nearby astrocytes: S100A10 in the treadmill group and C3d in the vehicle (Figure 1F). These results suggest that treadmill training increases mitochondrial dosage in the corpus callosum. This increase may protect against the progression of ischemic white matter injury and prevent the transformation of protective astrocytes into inflammatory astrocytes.

### 2.2 Training Affects Motor Function After Cerebral Ischemia of Distal MCAO

**FIGURE 2:**
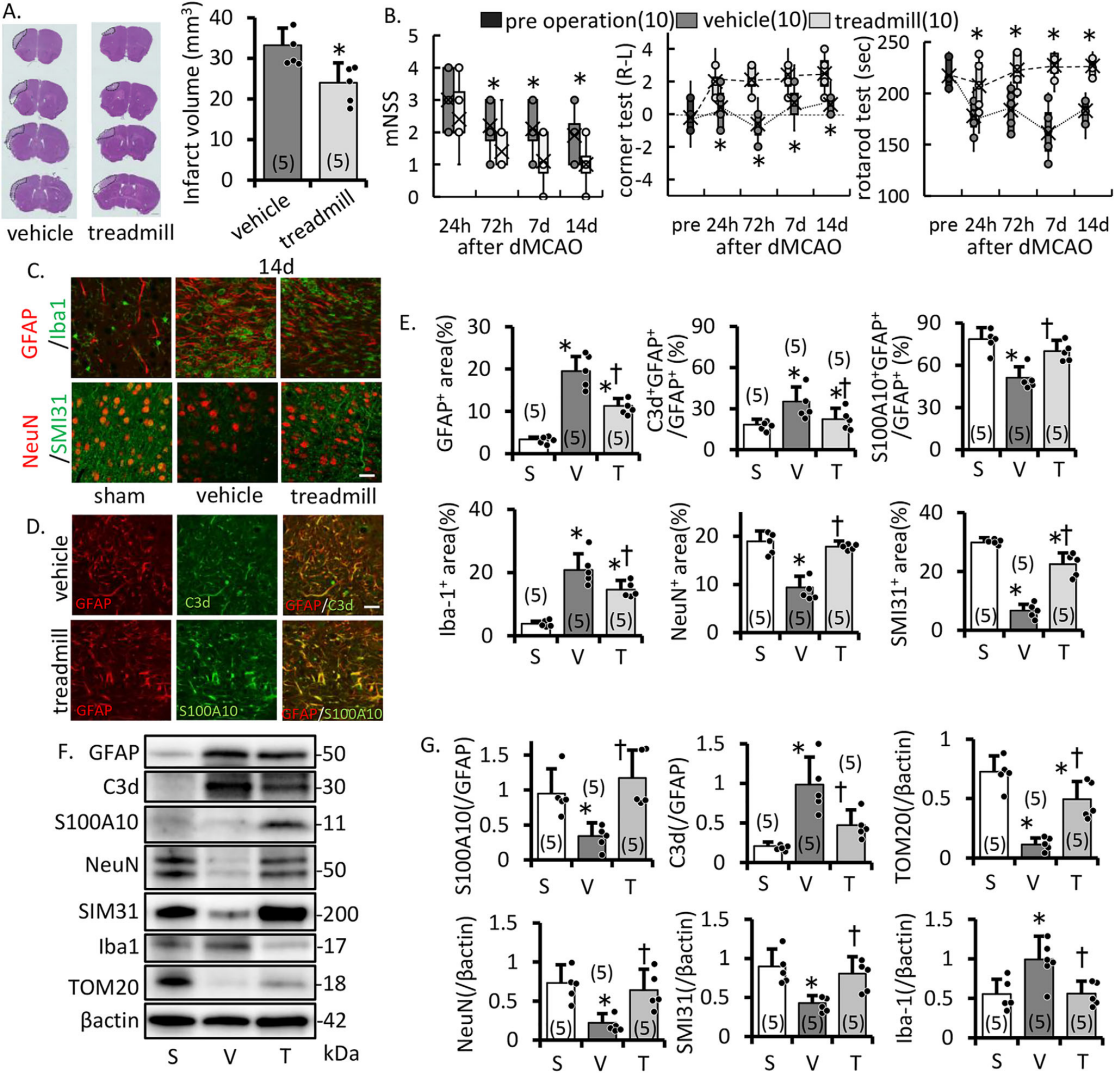
Effect of treadmill training for distal MCAO model. Serial changes in neuronal cell and astrocytes in periinfarct area. (A) HE stains and infarct volume at 14 days after dMCAO. (B) Motor function analysis of mNSS, corner test, and rotarod test after dMCAO. Treadmill training decreases infarct volume and post‐stroke sequelae. (C) Representative image and intensity of GFAP/Iba1 and NeuN/SMI31 at day 14 after operation. Scale bar, 50 µm. (D) Changes in astrocyte properties; GFAP (red), C3d (green), and S100A10 (green) immunostaining of periinfarct area at day 14 after dMCAO. Scale bar, 50 µm. (E) Intensity of GFAP, Iba1, NeuN, SMI31, and S100A10/C3d ratio of GFAP. Representative image (F) and densitometric analysis (G) of western blotting of the periinfarct area on day 14 after dMCAO. Treadmill training protects against astrogenesis, microglial activation, and neuronal loss, and maintains neurofilament number, myelin density, and protective astrocytes after acute ischemia. S, sham; V, vehicle; T, treadmill group. Data are the mean ± SD of *n* = 5, except B; *n* = 10. *p ≤ 0.05 compared to the sham group, †*p* ≤ 0.05 compared to the vehicle group.

We also surveyed the effect of treadmill training on post‐stroke motor function after distal MCAO. The infarct volume was smaller in the training group (Figure 2A). Furthermore, mice motor function, assessed through mNSS, rotarod test, and corner test, showed improvement in the training group (Figure 2B). In the periinfarct area, training suppressed microglial(Iba‐1^+^)/astroglial (GFAP^+^) activation and maintained neuronal structure (NeuN^+^ area; neuronal marker, SMI31^+^area; nerve axonal marker) (Figure 2C,E). Moreover, higher GFAP‐positive cells suppressed C3d^+^ inflammatory astrocytes and upregulated S100A10^+^ protective astrocytes (Figure 2D,E). Similar results to immunostaining were obtained by WB. Mitochondrial maker TOM20 band intensity in the periinfarct area was increased in the treadmill group (Figure 2F,G). These results suggest treadmill training decreases the sequelae of cerebral infarction, suppresses inflammatory cells, and activates protective cells.

### 2.3 Training Effect for Muscle Volume Quantity and Mitochondria Dosage in Blood

**FIGURE 3:**
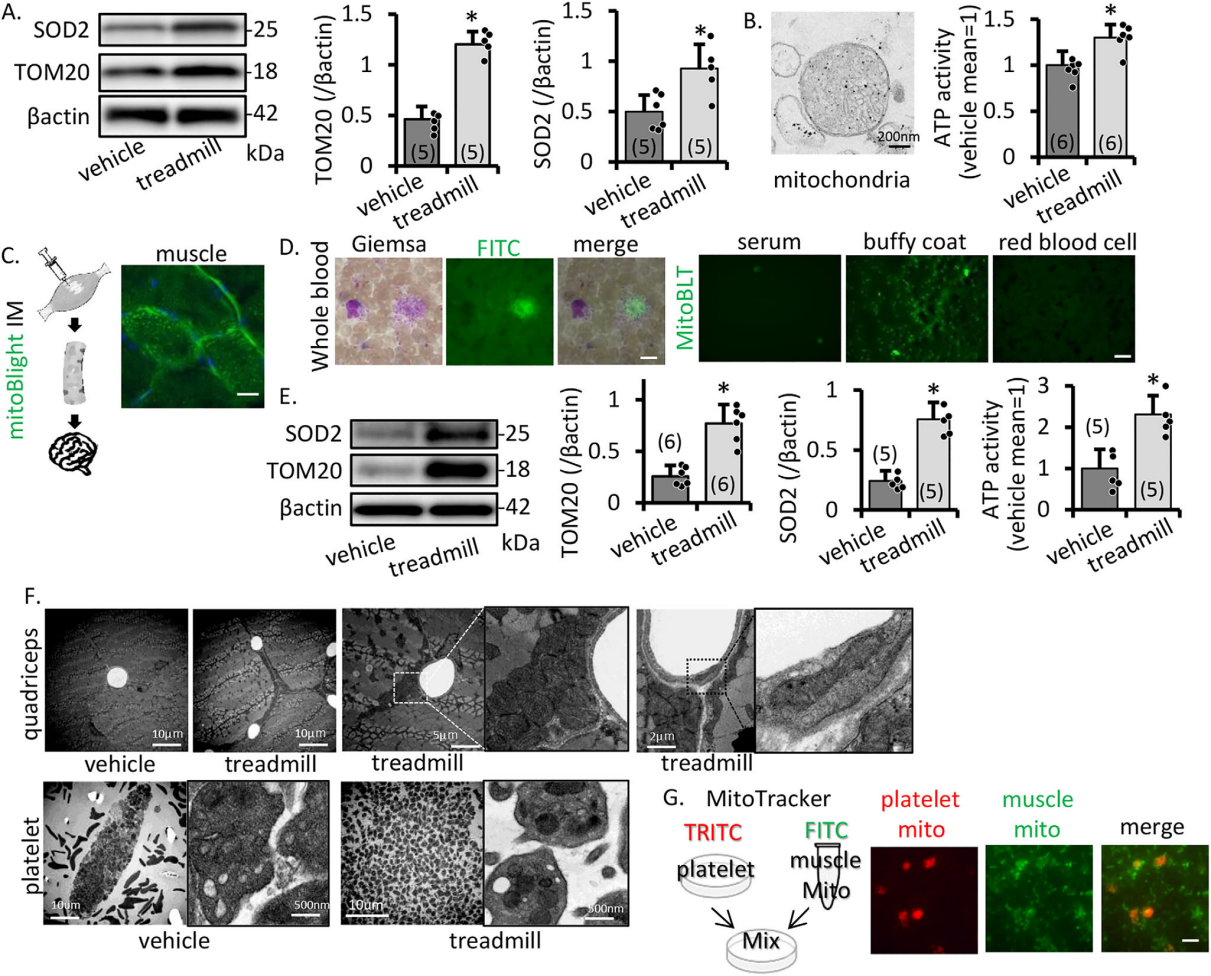
Effect of treadmill training for muscle/platelet mitochondria. (A) Representative image and intensity of TOM20 and SOD2 western blotting of the quadriceps after treadmill training. (B) Transmission electron microscope image of mitochondria, and mitochondrial ATP activity of same volume muscle. Mitochondrial number and activity are increased by training. (C) Representative schema and images of mitochondria labeling. Mitochondria have FTIC signal after Mitobilight Green injection. Scale bar, 100 µm. (D) FITC signal corresponds to the location of platelets on Giemsa stain (scale bar, 100 µm), and the green signal is seen in the buffy‐coat, not serum and red blood cells (scale bar, 100 µm). (E) Representative image, intensity of TOM20, SOD2 western blotting, and mitochondrial ATP activity of platelet after treadmill training. Mitochondrial number and activity are increased in platelets by training. (F) Transmission electron microscope image of quadriceps and platelets in the vehicle and treadmill groups. After training, mitochondria increase under fascia and increase in platelets. (G) Representative schema and images of mixed FITC‐MitoTracker‐labeled muscle mitochondria and TRITC‐labeled platelet at 3 h after mixture. Muscle mitochondria transferred into platelets. Scale bar, 25 µm. Data are the mean ± SD of *n* = 5–6. *p ≤ 0.05.

We investigated the origin of the increased mitochondria. Treadmill training increases skeletal muscle volume quantity. Muscle is associated with a high density of mitochondria [10]. Therefore, we focused on skeletal muscle mitochondria. In the WB analysis of quadriceps samples with equal volumes, the band density of mitochondrial maker TOM20 increased with treadmill training (Figure 3A). Transmission electron microscopy (TEM) revealed that the shape of the mitochondria from muscle was maintained in normal morphology. Mitochondrial ATP content was higher in the training group than in the vehicle group (Figure 3B).

We investigated how the transfer from the muscle to the brain occurred. For labeling muscle mitochondria, we used mitoBlight‐FITC by intramuscular injection for quadriceps. One day after injection, muscle mitochondria had FITC‐signals (Figure 3C). In whole blood, FITC signals were found in only platelets by Giemsa staining (Figure 3D). We separated whole blood into serum, buffy coat, and red blood cells by centrifugation and confirmed these phenomena by checking FITC signals. We found FITC in the buffy coat area (Figure 3D). This suggests that the mitochondria move by transporting platelets. Mitochondria dosage and activity (ATP‐activity) in platelets was higher in the treadmill group than the vehicle (Figure 3E). Moreover, TEM showed that the number of mitochondria in muscle and platelets increased. These mitochondria continued to function well in an oxygen‐rich environment. The mitochondria were clustered beneath the fascia and increased in platelets (Figure 3F).

To confirm that platelets take up mitochondria from the blood, we performed in vitro experiments. Platelet‐resident mitochondria were labeled with TRITC, to which FTIC‐labeled muscle‐derived mitochondria were added. It was checked 3 h later. The FITC signal was confirmed in the platelets, indicating that the mitochondria were transferred to the platelets (Figure 3G). To verify that these mitochondria were functional, we confirmed that SOD2 activity in platelets and muscle of the treadmill group (in this experiment, platelet was stored at −80°C until use) was increased compared to the vehicle (Figure 3A,E).

These findings suggest that functional mitochondria increased in muscle as a result of exercise, and subsequently also increased in platelets, indicating that muscle‐derived mitochondria may have been taken up by platelets.

### 2.4 Migration and Cellular Localization of Transferred Mitochondria in Ischemic Brain

**FIGURE 4:**
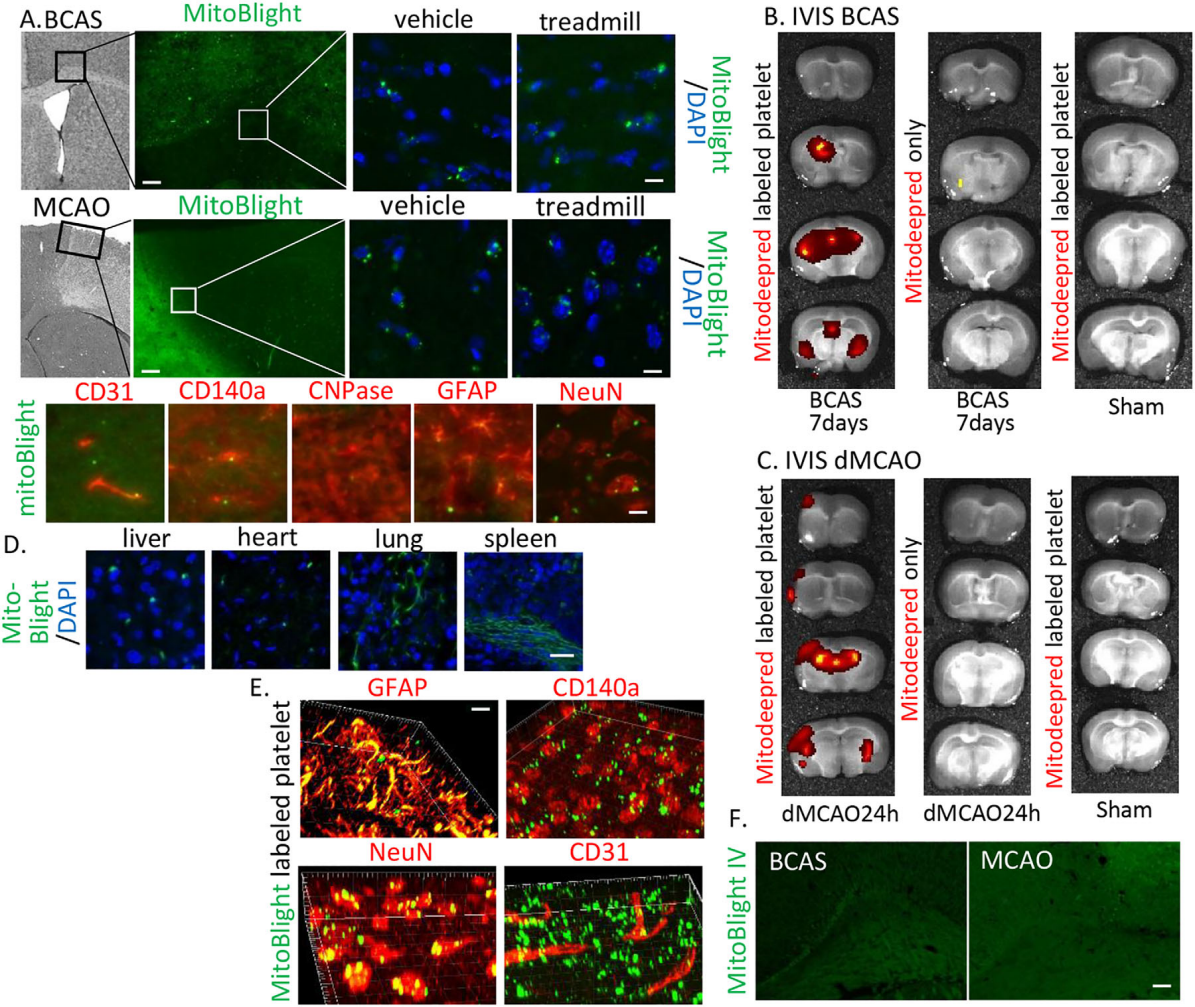
Mitochondrial migration into brain parenchyma after ischemia. (A) Representative image of corpus callosum/penumbra at 24 h after mitoBlight‐FITC injection into quadriceps after BCAS/dMCAO. FITC‐signal is found in corpus callosum at 7 days after BCAS operation (scale bar, left 200 µm/right 25 µm), and merged with CD31 (micro vessel), CD140a (OPC), GFAP (astrocyte), and NeuN (neuron). Scale bar = 25um. (B) IVIS image of brain in BCAS at 7 days and sham operation at 24 h after injection of MitoBlight‐deepred‐labeled platelet or MitoBlight‐deepred only. Red signal is seen in the damaged area of BCAS/labeled platelet group. (C) IVIS image of brain in dMCAO 24 h and sham operation at 24 h after injection of MitoBlight‐deepred‐labeled stored platelet or MitoBlight‐deepred only. Red signal is seen in damaged area of dMCAO/labeled platelet group. (D) Representative image of liver, heart, lung, and spleen at 24 h after mitoBlight‐FITC injection into quadriceps. FITC signal is found in each sample. Scale bar, 100 µm. (E) Z‐stack image of corpus callosum/penumbra at 24 h after mitoBlight‐FITC‐labeled stored platelet after BCAS 7 days/dMCAO 24 h. FITC‐signal was merged with CD31 (microvessel), CD140a (OPC), GFAP (astrocyte), and NeuN (neuron). Scale bar = 10 µm. (F) Representative image of corpus callosum/penumbra at 24 h after mitoBlight‐FITC intravenous injection. Scale bar, 100 µm. This experiment was repeated over three times, independently.

Next, we investigated whether the mitochondria increased in muscle and platelets through exercise were carried to the brain. FITC‐labeled mitochondria derived from injection into quadriceps were found in brain parenchyma (Figure 4A) and various organs including liver, heart, lung, and spleen (Figure 4D). In the corpus callosum, FITC signals merged with microvessels (CD31^+^ cells), oligodendrocyte progenitor cells (OPCs; CD140a^+^ cells), and astrocytes (GFAP^+^ cells) after BCAS (Figure 4A). In penumbra, a signal was found in microvessels, astrocytes, and neurons (NeuN^+^ cells) after MCAO (Figure 4A). For confirmation, we injected mitodeepred‐labeled stored platelets through the tail vein and observed them 24 h later using the IVIS system. Mitodeepred signal was observed in the white matter of the BCAS group (Figure 4B) and in periinfarct area of distal MCAO 24 h after mitodeepred ‐labeled stored platelets injection (Figure 4C). Nevertheless, no brain accumulation was detected by IVIS imaging following administration of MitoDeepRed solution alone or in the sham model. For further confirmation, MitoBlight‐FITC labeled stored platelets was injected after 24 h of BCAS 7day/MCAO 24 h. Green signal was found in astrocyte, OPC, neuron, and microvessels in brain ischemic lesion (Figure 4E, Z‐stack image). However, MitoBlight FITC injection did not identify FITC signal in the brain parenchyma (Figure 4F).

These results suggest that muscle training leads to an increase in muscle mitochondria, which subsequently migrate to the white matter/periinfarct area. The migration of mitochondria may play a protective role against white matter injury and cerebral infarction.

### 2.5 Mitochondrial Migration Effect for White Matter/Periinfarct Area Constitutes Cells In Vitro

To assess whether the beneficial effects of exercise could be recapitulated, platelets were isolated from treadmill‐trained rat and administered to ischemic models. This approach was based on the assumption that platelet transfusion represents the transfer of mitochondria that had increased within platelets as a result of exercise‐induced stress. The primary aim of this experiment was to evaluate the neuroprotective effects of mitochondria in the context of cerebral ischemia. We first conducted investigations using in vitro models.

**FIGURE 5:**
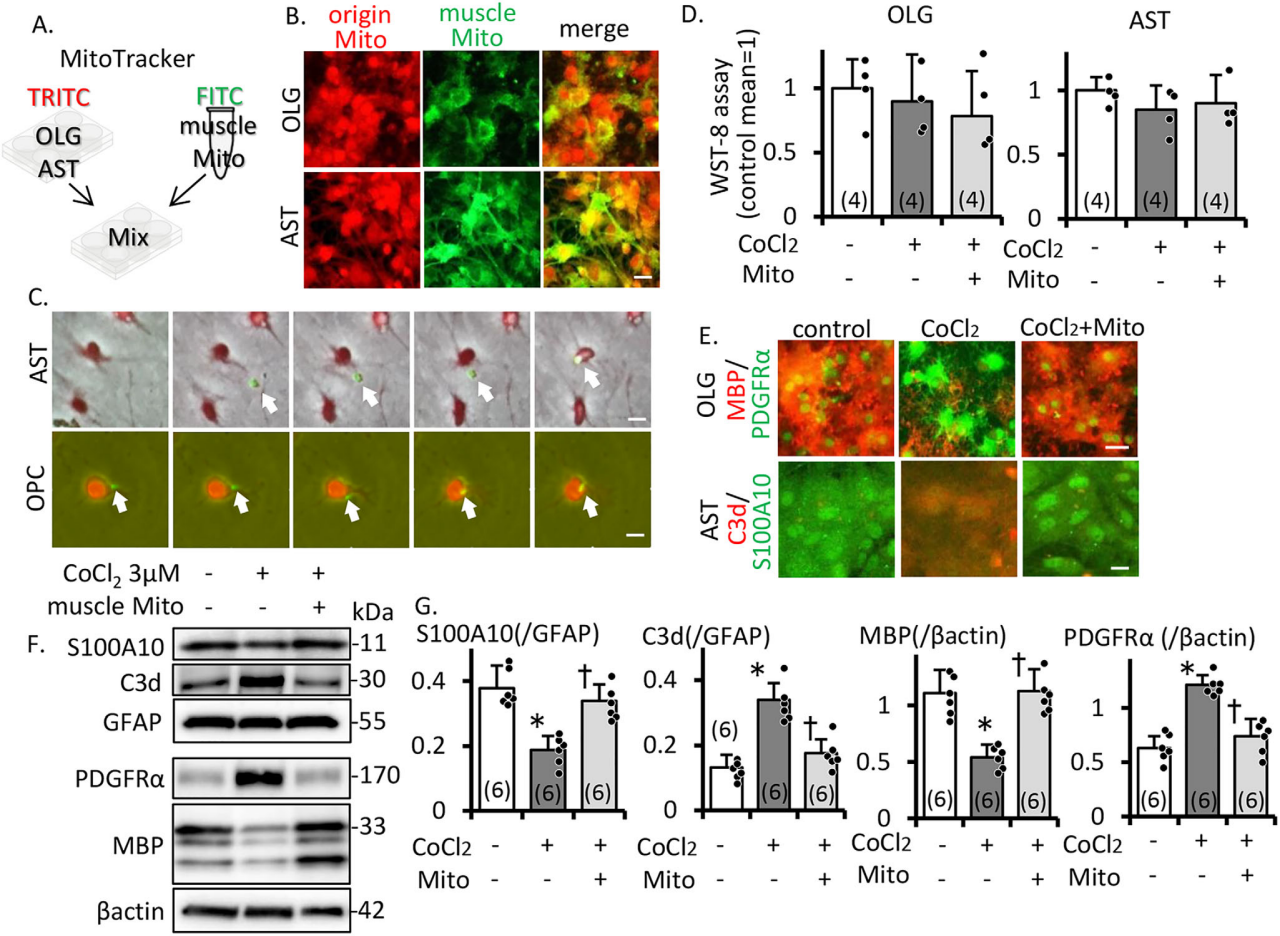
Effect of mitochondria on oligodendrocyte linage cells and astrocyte under mimic hypoperfusion in vitro. Representative schema (A) and images (B) of mixed FITC‐MitoTracker‐labeled muscle mitochondria and TRITC‐labeled OLG/AST at 3 h after mixture. OLG, oligodendrocytes; AST, astrocytes. Scale bar, 25 µm. Muscle mitochondria were transferred into OLG/AST. (C) Time‐lapse image of AST/OLG. Allow; FITC‐labeled mitochondria. Mitochondria migrate into cell body of AST/OLG. Scale bar, 20 µm. (D) WST assay of OLG/AST. Mitochondria administration was no effect for cell survival with/without CoCl_2_. (E) Immunocytochemistry of mitochondria‐treated OLG/AST. Scale bar, 25 µm. Western blotting (F) and densitometric analysis (G) of mitochondria treatment of OLG/AST cell lysate. β‐Actin is an internal control. Mitochondria‐treatment increased OPC maturation even after CoCl2 treatment and assist for maintaining the healthy type of AST. This experiment was repeated over three times, independently. Data are the mean ± SD of *n* = 4–6. *p ≤ 0.05 compared to control, †*p* ≤ 0.05 compared to CoCl2 and CoCl2+mitochondria treatment group.

For checking mitochondrial migration, resident mitochondria were labeled by TRITC‐MitoTracker, and muscle extract mitochondria were labeled with FITC‐MitoTracker (migrate mitochondria) and mixed (Figure 5A). After the mixture, OLG and astrocyte had green and red signals (Figure 5B). We recorded a time‐lapse movie (Figure 5C, Supporting Information: timelaps_OPCmito and timelaps_ASTmito). Mitochondria have both beneficial effects (energy storage) and harmful effects (oxidative stress). We investigated the mitochondrial migration effect. We found no significant difference in cell survival rate by WST assay in three groups of OLG linage cells/astrocyte (Figure 5D). Even under simulated hypoperfusion, immunocytochemistry and WB analysis suggested that the administration of mitochondria aided in the maturation of OLG lineage cells and prevented the transformation of protective astrocytes into inflammatory astrocytes (Figure 5D–F). These findings suggest that the migration of muscle mitochondria did not induce inflammation or enhance oxidative stress but rather assisted in maintaining the white matter environment in a normal condition, contributing to the support of protective astrocytes and the maturation of OLG lineage cells even in simulated hypoperfusion.

**FIGURE 6:**
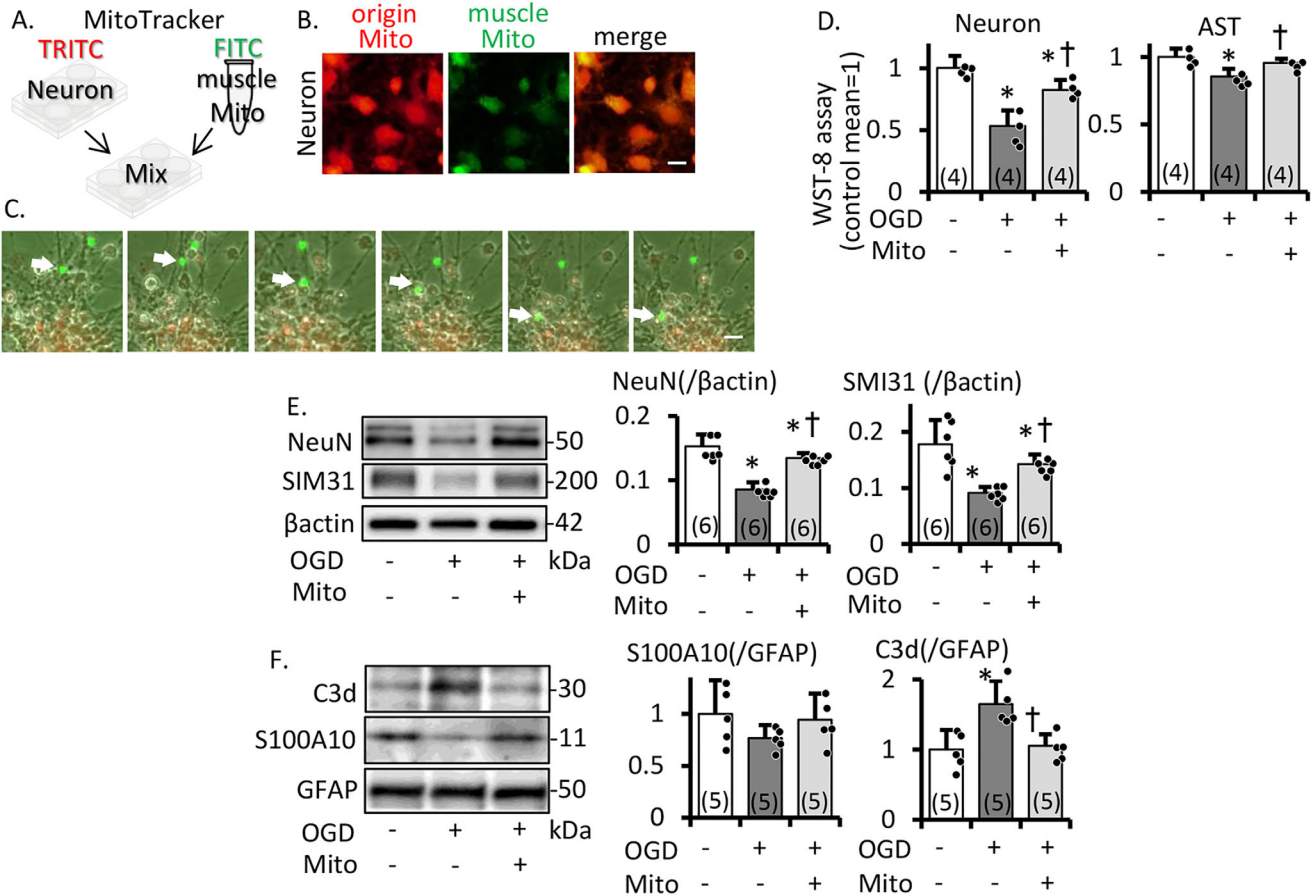
Effect of mitochondria on neuron and astrocyte under OGD in vitro. Representative schema (A) and image (B) of mixed FITC‐MitoTracker‐labeled muscle mitochondria and TRITC‐labeled neuron at 3 h after mixture. Scale bar, 25 µm. Muscle mitochondria were transferred into neuron. (C) Time‐lapse image of neuron. FITC‐labeled mitochondria. Mitochondria migrate into cell body of neuron. Scale bar, 50 µm. (D) WST assay of neuron/astrocyte. Mitochondria administration increases cell survival of neuron/astrocyte at 72 h after OGD. Western blotting and densitometric analysis of mitochondria treatment of neuron (E) and astrocyte (F) cell lysate. β‐Actin is an internal control. Mitochondria treatment maintains expression of NeuN/SMI31 on neuron, and decreases C3d/increased S100A10 expression on astrocytes at 72 h after OGD. This experiment was repeated over three times, independently. Mean ± SD of *n* = 5. *p ≤ 0.05 compared to control, †*p* ≤ 0.05 compared to OGD and OGD+mitochondria treatment group.

We also examined the effect of mitochondria transfer on neurons and astrocytes, aiming to mimic cells found in the periinfarct area. After the mixture of cultured neuron and muscle mitochondira, muscle mitochondria (green) were observed in neurons (Figure 6A–C, Supporting Information: timelaps_neuronmito). Under conditions simulating acute ischemia (OGD induction), mitochondria migration contributed to the survival of neuronal and astrocyte cells by WST‐8 analysis (Figure 6D), as well as the preservation of neuronal maker (NeuN^+^)/neurofilament marker (SMI31^+^) expression (Figure 6E). Furthermore, mitochondria migration suppressed the transformation of astrocytes from an inflammatory (C3d^+^) to a protective (S100A10^+^) state (Figure 6F). These results suggest that muscle mitochondria migration does not induce inflammation or enhance oxidative stress but rather assists in neuronal cell survival and neurofilament preservation, while maintaining astrocytic conditions.

### 2.6 Effects of Mitochondrial Treatment in the BCAS Model

**FIGURE 7:**
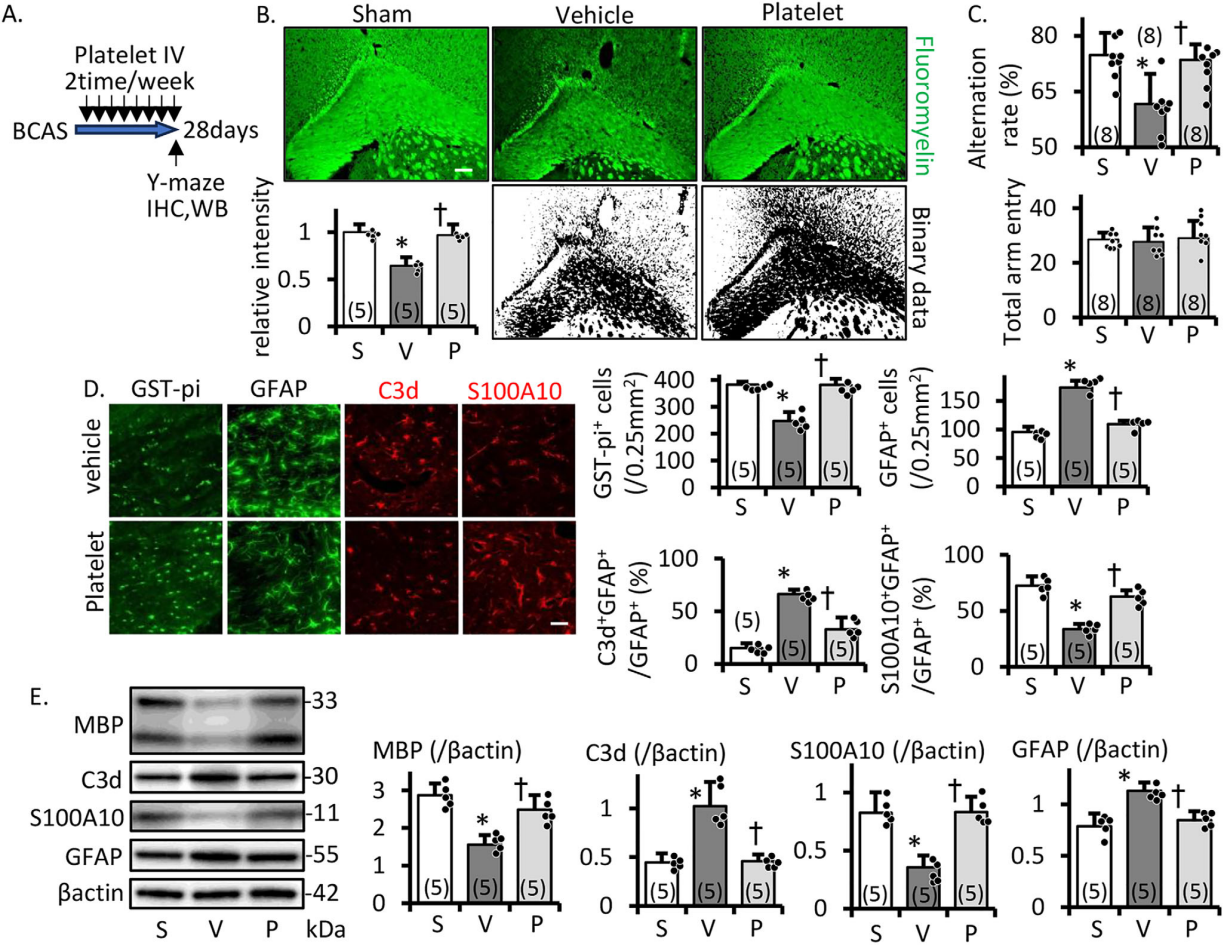
Effect of tPDM treatment in BCAS model. (A) Conceptual diagram of the experiment. Treadmill trained platelet derivate mitochondria (tPDM; equivalent with platelet dose 0.1 U/kg) injected two times per week. (B) FluoroMyelin staining and relative FluoroMyelin intensity in the corpus callosum at 28 days after BCAS operation. Scale bar, 100 µm. (C) Spatial memory assessed by spontaneous Y‐maze test; alternation rate and locomotor activity (total arm entry). tPDM protected against white matter damage progression and recent memory disturbance. (D) Representative image and intensity of GST‐pi (green), GFAP (green), C3d (red), and S100A10 (red) immunostaining of the cerebral corpus callosum on day 28 after BCAS. Scale bar, 50 µm. E. Representative image and densitometric analysis of Western blotting of the corpus callosum on day 28 after BCAS. tPDM protected astrogenesis and maintained oligodendrocyte number, myelin density, and protective astrocytes under hypoperfusion. S, sham; V, vehicle; P, tPDM treatment group. Mean ± SD of *n* = 5, except (C); *n* = 8. *p ≤ 0.05 compared to sham group, †*p* ≤ 0.05 vehicle versus tPDM treatment group.

We next analyzed the histochemical findings using mouse BCAS model to validate the in vitro experiments. First, using the prolonged cerebral hypoperfusion model mice, we confirmed the outcome of treadmill exercise for preconditioning mouse platelets. For making treadmill trained platelet derivate mitochondria (tPDM), platelets were isolated from treadmill trained mouse (8 m/min for 30 min three times a week for 2 weeks) of 0.7–0.8 mL whole blood, diluted in 1 mL of PBS (stored at −80°C until administration), and administered to stroke model mouse at a dose of 100 µL (approximately 0.1 unit/kg; human equivalent: 5 units/time). From our preliminary analysis, tPDM were injected into tail vein 2 times per week (data not shown). At 28 days after hypoperfusion, white matter lesions (fluoromyelin intensity) had progressed in the vehicle group, whereas platelet treatment reduced white matter damage (Figure 7A,B). Based on the Y‐maze test, spatial memory disturbance without locomotor disruption (arm entry) had progressed at 28 days after the operation in the vehicle treatment group. However, platelet treatment maintained the same degree of spatial memory as that in the sham group (Figure 7C). tPDM treatment maintained mature OLG (GST‐pi^+^) number (Figure 7D) and MBP intensity (Figure 7E). Regarding astrocyte expression, platelet treatment reduced GFAP intensity compared to the vehicle (Figure 7D). Furthermore, S100A10 (protective astrocyte maker)/C3d (inflammatory astrocyte maker) expression patterns were similar to those in the sham group (Figure 7D). These findings suggest that platelet‐derived mitochondria play a role in maintaining protective astrocytes under hypoperfusion conditions, and the migration of mitochondria in astrocytes and OPCs is similar, contributing to the protection of white matter homeostasis. Consequently, both white matter damage and memory disturbance were mitigated.

### 2.7 Effects of Mitochondrial Treatment in the Distal MCAO Model

**FIGURE 8:**
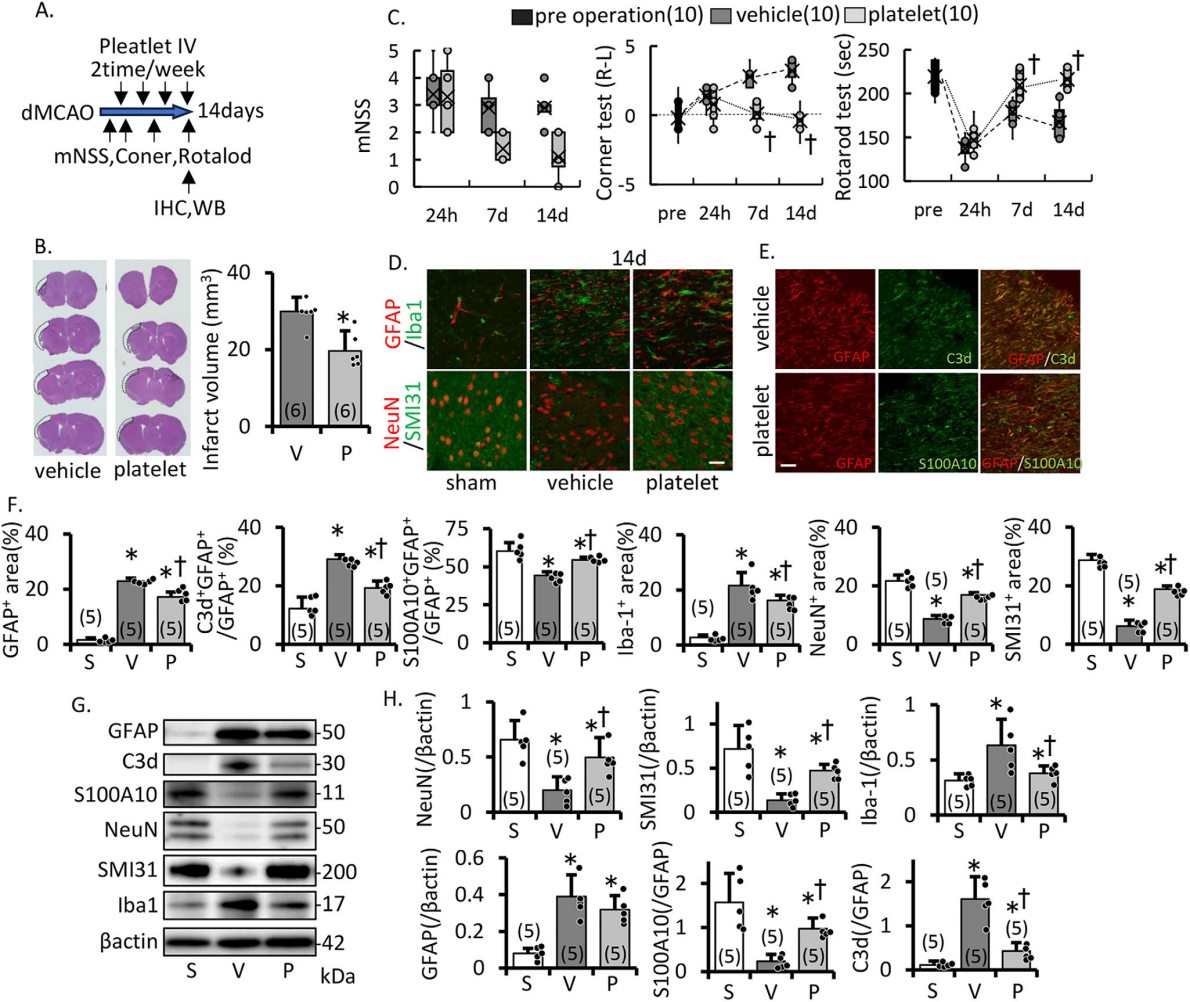
Effect of tPDM treatment for distal MCAO model. (A) Conceptual diagram of this experiment. Treadmill trained platelet derivate mitochondria (tPDM; equivalent with platelet dose 0.1 U/kg) injected two times per week from 24 h after operation. (B) HE staining and infarct volume at 14 days from dMCAO. tPDM treatment significantly decreases infarct volume. (C) Motor function analysis by mNSS, corner test, and rotarod test after dMCAO. tPDM treatment decreases post‐stroke sequelae. (D) Representative image and intensity of GFAP/Iba and NeuN/SMI31 at day 14 after operation. (E) Changes in astrocyte properties; GFAP (red), C3d (green), and S100A10 (green) immunostaining of periinfarct area at day 14 after dMCAO. Scale bar, 50 µm. (F) Intensity of GFAP, Iba1, NeuN, SMI31, and S100A10/C3d ratio of GFAP. Representative image (G) and densitometric analysis (H) of western blotting of the periinfarct area on day 14 after dMCAO. tPDM treatment protected against astrogenesis and microglial activation neuronal loss, and maintained neurofilament number, myelin density, and protective astrocytes after acute ischemia. S, sham; V, vehicle; P, tPDM treatment group. Mean ± SD of *n* = 5, except (C); *n* = 10. *p ≤ 0.05 compared to the sham group, †*p* ≤ 0.05 vehicle versus tPDM treatment group.

Next, using distal MCAO model mice, we confirmed the outcome of tPDM administration for acute ischemia (Figure 8A). At 14 days after MCAO, cerebral infarction volume was significantly decreased in the platelet treatment group compared to the vehicle (Figure 8B). The motor function of mice, assessed by mNSS, rotarod test, and corner test, showed greater improvement in the training group (Figure 8C). In the periinfarct area, platelet‐derived mitochondria treatment suppressed microglial (Iba‐1^+^)/astroglial (GFAP^+^) activation and maintained neuronal structure (NeuN^+^, SMI^+^ area) (Figure 8D,F). Furthermore, higher GFAP‐positive cells suppressed inflammatory astrocytes (C3d^+^) and upregulated protective astrocytes (S100A10^+^) in immunohistochemistry (Figure 8E,F). Similar results to immunostaining were obtained by WB (Figure 8G,H). These results suggest that platelet‐derived mitochondria decrease the sequelae of cerebral infarction, suppress inflammatory cells, and activate protective cells.

## 3 Discussion

Our findings suggest that increased intramuscular mitochondria are transferred to various organs by platelets under exercise stress, providing a protective effect in both chronic ischemia and the acute phase of the model. Platelets are activated by endothelial cell damage and release their contents, as confirmed by their aggregation at the site of brain damage in our experiments. While cytokines and other factors have been reported to influence exercise loading [22], there is currently no literature on the direct effect of exercise loading. Similarly, recent reports have highlighted the ischemic protection effect of remote ischemic loading, with a study indicating the involvement of extracellular vesicles [23]. However, the exact mechanism remains unclear. The association with muscle suggests that the transfer of mitochondria from muscle to the brain is involved in the process.

Recent studies have highlighted the prognostic significance of platelet count in various patient populations. In particular, abnormal platelet counts have been identified as poor prognostic factors in critically ill patients [24] and those with cancer [25, 26]. Additionally, platelet count has been associated with mortality in both elderly individuals and the general population [27, 28, 29]. One study reported a U‐shaped relationship between platelet count and mortality in older adults, with associations noted for both cancer‐related and cardiovascular mortality [30]. In the context of stroke, several platelet‐related factors—such as von Willebrand factor, CD40 ligand, and monocyte–platelet aggregates—have been implicated in poor functional outcomes based on evidence from both basic animal studies and human serum analyses [31, 32]. We have previously reported that both low and high platelet counts are associated with unfavorable functional outcomes at the time of discharge, as well as with early neurological deterioration in stroke patients [33]. Furthermore, prior research has shown that elevated platelet counts increase the risk of ischemic stroke [34], whereas patients with mid‐range platelet counts tend to have better long‐term functional outcomes during rehabilitation [35]. These findings suggest that maintaining platelet counts within the normal range is associated with the most favorable prognosis across various disease conditions.

Intercellular mitochondrial transfer has emerged as a novel paradigm of intercellular signaling [36, 37]. In the cardiovascular system, mitochondrial transplantation has been shown to protect cardiac muscle cells from ischemia–reperfusion injury [38]. Similarly, mitochondria derived from mesenchymal stem cells can migrate to damaged tissues, where they restore metabolic function and protect target cells following injury [39]. In the central nervous system, microglia and astrocytes have been reported to transfer mitochondria to neighboring injured neurons, promoting neuroprotection and cell survival [40, 41]. Astrocytes also transfer mitochondria to OLG lineage cells in injured cerebral white matter, thereby facilitating the proliferation and maturation of OLG precursor cells [12]. Endothelial progenitor cells may likewise engage in mitochondrial transfer to support and protect the brain endothelium [42]. Mitochondria have also been observed to be released into the extracellular space under specific conditions [43]. For instance, retinal neurons can release mitochondria, which are then taken up by optic nerve head astrocytes for recycling and disposal [44]. Likewise, mitochondria from bone marrow–derived stromal cells can migrate to the alveoli to mitigate acute lung injury [39]. In the brain, xenogeneic mitochondrial transplantation has been shown to improve long‐term outcomes in a rat model of focal cerebral ischemia by enhancing glucose metabolism at the injury site [45]. Furthermore, direct intravenous administration of mitochondria has demonstrated potential in promoting functional recovery after intracerebral hemorrhage [46] and in alleviating age‐related cognitive decline [47].

A previous study reported the importance of the cell–cell interaction neuro‐oligo‐vascular niche in brain tissue [48]. “Help‐me signal”‐mediated interactions occur between dysfunctional cells and cells that maintain normal function in tissues [49]. Although various candidate factors responsible for the interaction have been reported, their molecular mechanisms are unknown, and no factors have been found to be clinically helpful. Single trophic factor supplementation has little impact on functional and disability recovery, and a previous study reported that it is ineffective in clinical practice [50]. Recently, the concept of secretome has been proposed, and “trophic coupling,” the interaction of multiple types of trophic factors between cells, is involved in regulating cellular functions [50]. From this, it can be inferred that mitochondria, rich in cAMP, may play a role in cell protection and functional recovery by acting as a direct trophic factor in therapy [51].

Although our results are a proof‐of‐concept study, there are several limitations. First, the molecular mechanism of mitochondrial release‐uptake remains unknown. Regarding mitochondrial uptake, our analysis was mainly based on media transfer; however, integrin‐mediated endocytosis [52] and micropinocytosis [53] may be involved in mitochondria internalization. Further studies are necessary to identify the distribution of mitochondria within such extracellular particles. And the mechanism by which mitochondrial uptake contributes to the restoration of impaired cellular function remains unclear, particularly whether this effect is mediated solely through bioenergetic support. However, in our in vitro model, mitochondrial administration led to increased cell viability, and even under chronic ischemic conditions, the treatment preserved astrocyte and OLG lineage cells. To provide direct evidence that muscle‐derived mitochondria are taken up by platelets and subsequently transported to neuronal cells, future experiments using transgenic mice that express mitochondria‐targeted fluorescent tracer proteins specifically in muscle cells would be necessary. Such models would enable the tracking of mitochondria following cerebral ischemia. However, a limitation of the present study is that we employed CB17 mice, a strain with limited applicability and limited precedent for genetic modification. Further investigations are warranted to elucidate the underlying mechanisms. Second, extracellular vesicles (also known as microparticles, microvesicles, and exosomes) have been examined as significant important contributing factors in cell–cell communication [49]. Extracellular vesicles comprise a lipid bilayer enclosing proteins and RNA that change the functions and state of the recipient cells by stimulating signaling via receptor‐ligand delivery or incorporating their message into the recipient cells [54]. Additional research is necessary to elucidate the mechanisms and potential roles of extracellular mitochondria in mediating cellular interactions. Third, we did not include platelets from the control group in the present study. We assessed mitochondrial content and ATP activity in the comparative analysis of platelets; thus, the impact of this omission may be limited. Nevertheless, it is conceivable that ATP activity within platelets may be diminished in the context of diabetes and other disease models [55]. Additional experiments comparing disease models and the normal group are a future challenge. Fourth, further studies are needed to determine the efficacy of this treatment under antiplatelet therapy, because some types of antiplatelet therapy are performed for secondary prevention of cerebral ischemia. Fifth, treadmill training might be affected by cerebral collateral flow. In this regard, for analysis, we checked vessel diameter at ACA‐MCA anastomosis, but there were no differences (Figure S1). However, this experiment could only surface, we could not check blood flow in deep white matter. And also, in order to check how long the effects of the exercise load would last, we checked the number of mitochondria and SOD2 activity in the platelets 2 or 3 weeks after the exercise load, but the effects of the exercise load disappeared after 3 weeks (data not shown). Sixth, treadmill‐trained platelet derivate mitochondria were administered twice a week in this experiment. Since platelets are supposed to be activated once they are refrigerated, platelets have a lifespan of 2 weeks and transfused platelets have a lifespan of 3–4 days [56]. In this context, it has been clinically confirmed that platelet counts can usually be maintained for 10 units/week or 20 units/2 weeks when administered to a person with thrombocytopenia. Our treatment uses stored platelets, although this retention period is not strictly consistent. The advantage of this therapy is that mitochondria can be extracted from platelets that could not be used for transfusion and used for treatment.

## 4 Conclusion

In this study, mitochondria were considered as a type of secretome, a group of cell‐secreted factors deeply involved in the regulation of cell–cell interactions. Mitochondria released from donor cells and taken up by recipient cells, together with regulated intercellular mitochondrial trafficking and dynamics, contribute to ischemic pathological processes. However, the protective effects of remote ischemic preconditioning, a phenomenon observed in recent years, may involve the mitochondria derived from these muscles. Furthermore, moderate rehabilitation after stroke may be involved in functional recovery. Although there have been recent setbacks in clinical trials of cell therapy, there is the potential for it to become an effective treatment. Our findings suggest the cytoprotective effects of the administration of mitochondria and their potential use as a novel treatment for post‐ischemic cerebral infarction in acute stroke patients and ischemic white matter damage in patients with vascular dementia.

## 5 Materials and Methods

### 5.1 Animal Models

All animal experiments were approved by the Animal Care Committee of Juntendo University and conducted in accordance with the National Institutes of Health Guide for the Care and Use of Laboratory Animals. Mice were housed under standard conditions with ad libitum access to food and tap water. Ten‐week‐old male C57BL/6 mice (Oriental Yeast Co., Ltd.) were used to establish a chronic cerebral hypoperfusion model. Bilateral common carotid artery stenosis (BCAS) was induced by placing micro‐coils (0.18 mm diameter; Sawane Spring) around both common carotid arteries, as previously described [57, 58]. For the acute cerebral ischemia model, 8‐ to 10‐week‐old CB17/Icr +/+ mice (Clea Japan, Inc.) were subjected to a modified Tamura method [59]. During the first 7 days post‐stroke, animals were provided with powdered chow and sterilized water to support recovery. To investigate the effects of preconditioning exercise, mice underwent treadmill training at 8 m/min for 30 min, three times per week, over a 2‐week period prior to the surgical procedure, till sacrificed. The exercise intensity was carefully selected based on previous findings indicating the superior physiological benefits of voluntary or mild exercise training [60]. Furthermore, this protocol was intentionally designed to be milder than those used in earlier studies, with preliminary experiments confirming that even this low‐intensity regimen was sufficient to promote measurable increases in muscle mass [61]. The platelet‐derived mitochondrial treatment group received mitochondria equivalent to a platelet dose of 0.1 unit/kg, administered intravenously twice per week. Cerebral infarct area was assessed using hematoxylin and eosin (H&E) staining, as previously described [59]. All in vivo experiments and outcome measurements were conducted in a randomized and blinded manner. In both the chronic and acute ischemia models, mice were randomly assigned to one of the following five groups: (1) ischemia‐only group (*n* = 12), (2) treadmill‐trained ischemia group (*n* = 12), (3) sham‐operated group (*n* = 24), (4) ischemia + platelet‐derived mitochondrial treatment group (*n* = 12), and (5) ischemia + vehicle treatment group (*n* = 12).

The inclusion criteria were the absence of focal foci of infarction in the corpus callosum in chronic ischemia and incomplete ischemia of the middle cerebral artery in acute ischemia. If, after ischemia, the animal showed more than 30% of its preoperative weight loss, abnormal behaviors such as circling and convulsions, or persistent lying down or cowering, it was euthanized and excluded from the experimental data. However, all mice survived and included experimental data.

### 5.2 Platelet Isolation and Muscle Mitochondria Isolation

We performed mitochondrial labeling in platelets. Whole blood samples were collected using pluriMate 2‐mL centrifuge tubes (pluriSelect Life Science; 44‐00002‐10) and PLT Spin Medium (pluriSelect Life Science UG & Co. KG; 60‐00094‐10). Giemsa staining was performed in some blood samples. Platelets were isolated according to the manufacturer's protocol. Platelets were stored at −80°C until administration. Platelets were isolated from 0.7 to 0.8 mL of treadmill trained mice (8 m/min for 30 min three times a week for 2 weeks) or 7 mL of treadmill trained rat (8 m/min for 30 min three times a week for 2 weeks) whole blood, diluted in 1 mL of PBS, and administered at a dose of 100 µL (approximately 0.1 unit/kg; human equivalent: 5 units/time). The same volume of platelet dilution solution was added to each well for cell culture. To extract mitochondria from muscle, mouse skeletal muscle was harvested under deep anesthesia. A portion was fixed with 2.5% glutaraldehyde and prepared for TEM. After deep freezing at −80°C, they were crushed into powder. The pellet was then purified using a mitochondria isolation kit (Mitochondria Isolation Kit, Bio Vision, Inc., K288‐50). Two groups, a treadmill and a normal group, were created (*n* = 10 in each group). Mitochondria were administered at a concentration of 0.1 mg/mL based on our preliminary experiments.

### 5.3 Assessment of Collateral Blood Flow

To evaluate exercise‐induced differences in collateral circulation, leptomeningeal anastomoses were visualized using the latex perfusion technique, as previously described [62]. Following transcardial perfusion with 20 mL of saline, 2.0 mL of a white latex compound (Product No. 563; Chicago Latex Products Inc., IL, USA), mixed with carbon black at a ratio of latex:PBS:carbon black = 10:6:4, was injected. Brains were then carefully extracted and fixed in 4% paraformaldehyde. Photographs of the dorsal surface of the brain were taken, and the diameters of leptomeningeal anastomoses were measured at five predefined points where the distal branches of the middle cerebral artery and anterior cerebral artery converge.

### 5.4 Y‐Maze Test

Spatial working memory was assessed using an 8‐min spontaneous alternation Y‐maze test conducted 28 days after BCAS, in the early morning, as previously described [12]. Each experimental group consisted of eight mice (*n* = 8). Briefly, each mouse was placed in a Y‐maze apparatus (arm dimensions: 40 cm in length, 13.5 cm in height, and 4 cm in width; Muromachi Kikai, Tokyo, Japan). The spontaneous alternation ratio was calculated as the number of actual alternations divided by the number of possible alternations, expressed as a percentage:

$$\begin{array}{c} \left(\text{Number of spontaneous alternations}/\right.\\ \left.\left[\text{Total number of arm entries}\;-\;2\right]\right)\times 100. \end{array}$$

In addition, the total number of arm entries during the session was recorded to evaluate locomotor activity.

### 5.5 Symptom Assessment After Acute Infarction

Symptoms after acute infarction were assessed by the following 3 methods [59]: *Modified neurological severity score (mNSS)*: An integrated neurological function score that combines motor, sensory, balance, and response tests, with a score of 0 for normal and 18 for maximum neurological function deficit. *Rotarod test*: A motor function test in which motor function is evaluated by recording the time (in seconds) it takes for the rodent model to fall from a rotating rotor. *Corner test*: Mice are placed in a corner with an angle of 30° and the direction in which they turn (right turn or left turn) when they reach the corner after performing exploratory behaviors is recorded. The test is repeated at least 10 times with an interval of at least 30 s within 30 min, and the number of [(right turn) − (left turn)] is calculated.

### 5.6 Immunofluorescence

Prior to transcardial perfusion, mice were deeply anesthetized with pentobarbital. Brains were immediately removed en bloc and post‐fixed in 4% paraformaldehyde in PBS at 4°C for 24 h. Following fixation, tissues were cryoprotected in 30% sucrose for at least 24 h. Brains were then frozen, and consecutive 16‐µm‐thick coronal sections were prepared using a cryostat (CM1900, Leica Instruments, Nussloch, Germany). Sections were blocked with 2% Blockace (Dainippon Sumitomo Pharma, Osaka, Japan; UK‐B80) for 1 h at room temperature, followed by overnight incubation at 4°C with primary antibodies. The following primary antibodies were used: mouse monoclonal anti‐GFAP (astrocyte marker; 1:200, Santa Cruz Biotechnology; sc‐33673), rabbit polyclonal anti‐GST‐pi (OLG marker; 1:200, MBL; code 312), mouse monoclonal anti‐Olig1 (OLG marker; 1:100, Millipore; MAB5540), mouse monoclonal anti‐CNPase (OLG marker; 1:100, Abcam; ab6319), rabbit polyclonal anti‐PDGFRα (OPC marker; 1:100, Santa Cruz Biotechnology; sc‐338), mouse monoclonal anti‐PDGFRα (1:100, Santa Cruz Biotechnology; sc‐398206), mouse monoclonal anti‐CD140a (OPC marker; 1:100, BD Biosciences; 558774), goat polyclonal anti‐C3d (inflammatory astrocyte marker; 1:50, R&D Systems; AF2655), rabbit polyclonal anti‐S100A10 (protective astrocyte marker; 1:100, Proteintech; 11250‐1‐AP), mouse monoclonal anti‐NeuN (neuronal marker; 1:100, Millipore; MAB377), mouse monoclonal anti‐SMI31 (axonal marker; 1:100, BioLegend; 801601), rabbit polyclonal anti‐Iba1 (microglial marker; 1:200, Wako; 019–19741), rat monoclonal anti‐CD31 (endothelial marker; 1:100, BD Pharmingen; 550274), and mouse monoclonal anti‐TOM20 (mitochondrial marker; 1:100, BD Transduction Laboratories; 612278). After washing with PBS, sections were incubated with appropriate secondary antibodies (1:250, Jackson ImmunoResearch Laboratories) for 1 h at room temperature, followed by mounting with VECTASHIELD containing DAPI (Vector Laboratories; H‐1200). Semi‐quantitative analysis was performed using 10× magnification images. Binary image conversion was applied to quantify the percentage of stained area relative to the total area, or to count the number of positively stained cells within a 0.25 mm^2^ region. For the prolonged hypoperfusion model, a blinded investigator counted positively stained cells in the lateral corpus callosum at bregma levels 1.18 mm, 0.98 mm, and 0.74 mm. For the acute ischemia model, three randomly selected regions within the peri‐infarct zone—located 300 µm from the ischemic core in cortical layer VI—were analyzed per section using a 20× objective.

### 5.7 FluoroMyelin Staining

Twelve‐micrometer‐thick coronal brain sections (bregma 0.86–0.50 mm) were incubated with FluoroMyelin Green fluorescent myelin stain (1:300; Molecular Probes; F34651) for 20 min at room temperature. Semi‐quantitative analysis of myelin staining intensity was performed using images acquired at 4× magnification. Images were converted to binary format using ImageJ software, and the mean fluorescence intensity was measured across the entire field of view for three sections per animal.

### 5.8 Cell Culture

Primary astrocytes, OPCs, and OLGs were isolated from the cerebral cortices of 1‐ to 2‐day‐old Sprague Dawley rats (Oriental Yeast Co., Ltd.) as previously described [58]. Briefly, cortices were dissected and enzymatically dissociated. Dissociated cells were plated into poly‐D‐lysine (Sigma; P6407)‐coated 75‐cm^2^ flasks and cultured in DMEM supplemented with 20% heat‐inactivated fetal bovine serum and 1% penicillin/streptomycin. After reaching confluency (approximately 10 days), microglia were removed by shaking the flasks for 1 h at 200 rpm and 37°C. Following a medium change, the flasks were shaken overnight to further eliminate microglia. To isolate OPCs, the medium was transferred to poly‐D‐lysine‐coated plates and cultured in neurobasal medium (Gibco, Grand Island, NY; 21103049) supplemented with glutamine, 1% penicillin/streptomycin (Gibco, Grand Island, NY; 15140122), 10 ng/mL PDGF (PeproTech; 100–13A), 10 ng/mL FGF (PeproTech; 400–29), and 2% B27 plus supplement (Gibco; 17504‐044). After 4–5 days, OPCs were collected for experiments. For astrocyte cultures, adherent cells were dissociated using trypsin and reseeded in poly‐D‐lysine‐coated flasks. Three hours after seeding, the flasks were shaken overnight at 200 rpm and 37°C, and the medium was replaced to collect purified astrocytes. Cells were used for experiments upon reaching 70%–80% confluency. To differentiate OPCs into myelin basic protein‐positive OLGs, cells were cultured in DMEM (Gibco; 11995‐073) containing 1% penicillin/streptomycin, 10 ng/mL CNTF (Sigma; 450‐50), 50 ng/mL T3 (Sigma‐Aldrich; T2877), and 2% B27 plus supplement.

Cortical neurons were obtained from embryonic day 17 Wistar rats (Charles River) following established protocols [63]. Dissociated neurons were plated onto poly‐D‐lysine‐coated plates at a density of 3 × 10^7^ cells/mL and maintained in neurobasal medium supplemented with 2% B27 supplement and 1% penicillin/streptomycin. To suppress astrocyte proliferation, uridine and 5‐fluorodeoxyuridine were added for 6 days.

To model chronic mild hypoxia, OPCs and astrocytes were treated with 3 µM cobalt chloride (CoCl_2_, Sigma; 7646‐79‐9) for 7 days [58]. The oxygen–glucose deprivation (OGD) model was deemed unsuitable for chronic ischemia, as it represents only a transient deprivation phase. In contrast, chronic cerebral hypoperfusion—as observed in the MCAO model with partial reperfusion in the peri‐infarct zone—was better replicated by sustained mild hypoxic stress using the CoCl_2_ model, allowing for consistent ischemic conditions in the cerebral white matter between experimental phases.

To model acute cerebral ischemia, cultured neurons and astrocytes were subjected to OGD. Briefly, culture medium was replaced with Ca^2^^+^‐ and Mg^2^^+^‐free Hank's balanced salt solution (Gibco; 14185‐052), and cells were incubated in a hypoxic chamber (5% CO_2_/95% N_2_) for 3 h. After OGD, cells were randomly assigned to experimental groups and returned to their original medium for 96 h.

Mitochondria or platelets were administered at concentrations determined in preliminary experiments. All in vitro experiments were performed in duplicate and independently repeated three to five times.

### 5.9 Immunocytochemistry

Cells were washed with PBS and fixed with 4% paraformaldehyde for 15 min at room temperature. After additional PBS washes, cells were blocked with 2% Blockace in PBS for 1 h. The following primary antibodies were used: goat polyclonal anti‐C3d (1:50; R&D Systems; AF2655), rabbit polyclonal anti‐S100A10 (1:100; Proteintech; 11250‐1‐AP), rabbit polyclonal anti‐PDGFRα (1:100; Santa Cruz Biotechnology; sc‐338), and mouse monoclonal anti‐myelin basic protein (MBP; OLG marker, 1:50; Millipore; MAB382). Following incubation with primary antibodies, cells were washed with PBS containing 0.2% Tween‐20 and incubated with appropriate secondary antibodies (1:200; Jackson ImmunoResearch Laboratories) for 1 h at room temperature. Slides were mounted using VECTASHIELD mounting medium with or without DAPI (Vector Laboratories; H‐1200).

### 5.10 Western Blotting

Cells from in vitro cultures were lysed in Cell Lytic Mammalian Tissue Lysis/Extraction Reagent (Sigma‐Aldrich; C3228). For tissue samples, mice were deeply anesthetized with isoflurane and transcardially perfused with 20 mL of PBS. Brains were rapidly removed and placed ventral‐side down in a brain matrix on ice. The brain was sectioned into three 1‐mm‐thick coronal slices, starting 2 mm posterior to the olfactory bulb. In the prolonged hypoperfusion model, the corpus callosum was carefully dissected on ice under a microscope using fine forceps. For the acute ischemia model, tissue was harvested from the ischemic cortical region. Samples were collected into microtubes, lysed in buffer, and homogenized using an ultrasonic disruptor. The following primary antibodies were used: mouse monoclonal anti‐GFAP (1:2000; Santa Cruz Biotechnology; sc‐33673), goat polyclonal anti‐C3d (1:1000; R&D Systems; AF2655), rabbit polyclonal anti‐S100A10 (1:2000; Proteintech; 11250‐1‐AP), mouse monoclonal anti‐MBP (1:2000; Thermo Scientific; MAB382), rabbit polyclonal anti‐PDGFRα (1:2000; Santa Cruz Biotechnology; sc‐398206), mouse monoclonal anti‐TOM20 (1:1000; BD Transduction Laboratories; 612278), rabbit polyclonal anti‐SOD2 (1:5000; proteintech; 24127‐1‐AP), mouse monoclonal anti‐NeuN (neuronal marker, 1:2000; Millipore; MAB377), mouse monoclonal anti‐SMI31 (axonal marker, 1:2000; BioLegend; 801601), rabbit polyclonal anti‐Iba‐1 (1:2000; Wako; 019–19741), and mouse monoclonal anti‐β‐actin (1:10000; Abcam; ab8226). Following overnight incubation at 4°C with primary antibodies, membranes were washed and incubated with HRP‐conjugated secondary antibodies (1:5000; Santa Cruz Biotechnology). Detection was performed using enhanced chemiluminescence (Amersham Biosciences).

### 5.11 ATP Measurement

Intracellular and extracellular ATP levels were measured using the CellTiter‐Glo Luminescent Cell Viability Assay (Promega, G7570), which induces cell lysis and generates a luminescent signal proportional to the ATP concentration. Briefly, 96‐well opaque‐walled plates containing 50 µL of test media per well were prepared. An equal volume (50 µL) of CellTiter‐Glo reagent was added to each well, followed by incubation for 30 min at room temperature. Luminescence was measured using a luminescence microplate reader [52].

### 5.12 Mitochondrial Labeling

For analysis of mitochondrial migration, mitochondria were labeled using 100 nM of MitoTracker Green FM (Invitrogen; M7514), MitoTracker Orange CMTMRos (Invitrogen; M7510), MitoBright LT Green (Dojindo; MT10), or MitoBright LT Red (Dojindo; MT11), according to the manufacturers' protocols. After two washes with PBS, cells were incubated in serum‐free medium. After 24 h, both cells and medium were collected for further analysis. For muscle‐derived mitochondrial labeling, 100 µL of MitoBright LT Green (Dojindo; MT10) was injected into the thigh muscles of mice. Mitochondria isolated from platelets were labeled with MitoBright LT Red (Dojindo; MT11) and injected intravenously (100 µL via the tail vein). After 24 h, mice were euthanized by decapitation, and fluorescence signals were confirmed using the IVIS imaging system.

### 5.13 Time‐Lapse Microscopic Analysis

Mitochondrial uptake into various cells was evaluated using a BioStation CT (Nikon, Tokyo, Japan) incubator equipped with a camera for video imaging. Phase contrast images were taken every 30 min for 300 min under brightfield conditions with a 20× lens.

### 5.14 Statistical Analysis

Power calculations were conducted with α = 0.05 and β = 0.8 to determine appropriate group sizes, targeting effect sizes in the range of 30%–50% for in vivo models and 40%–50% for cell culture experiments. For all parametric tests, data were assessed for normality and homogeneity of variances. An unpaired *t*‐test was used to compare differences between two groups. For comparisons involving more than two groups, continuous variables were analyzed using one‐way ANOVA followed by Tukey's honestly significant difference (HSD) test. Non‐parametric variables were analyzed using the Kruskal–Wallis test followed by appropriate multiple comparison procedures. Data are presented as mean ± standard deviation (SD), and a *p*‐value of ≤ 0.05 was considered statistically significant.

## Author Contributions

*Conceptualization*: T.I., N.M., and Y.U. *Methodology*: T.I. and N.M. *Investigation*: T.I., N.M., K.H., C.K., Y.M., H.X., and K.K. *Formal analysis*: T.I. and N.M. *Resources*: N.M., Y.U., and N.H. *Writing – original draft*: N.M. *Writing – review and editing*: All authors. Supervision: Y.U. and N.H. All authors have read and approved the final manuscript.

## Funding

This study was supported by JSPS KAKENHI (JP22K07355 and JP25K12513) and partly by (i) a grant from the High Technology Research Center, (ii) a grant‐in‐aid for exploratory research from the Ministry of Education, Culture, Sports, Science and Technology, Japan, and (iii) grants‐in‐aid from the Foundation of Strategic Research Projects in Private Universities from the Ministry of Education, Culture, Sports, Science, and Technology.

## Ethics Statement

All experimental procedures were approved by the Animal Care Committee of Juntendo University (No. 1301 and 1536).

## Conflicts of Interest

The authors declare no conflicts of interest.

## Supporting information




**Supporting File 1**: mco270590‐sup‐0001‐SuppMat.docx.

**Supporting File 2**: mco270590‐sup‐0002‐VideoS1.mp4.

**Supporting File 3**: mco270590‐sup‐0003‐VideoS2.mp4.

**Supporting File 4**: mco270590‐sup‐0004‐VideoS3.mp4.

## Data Availability

The datasets that support the findings of this study are available from the corresponding author upon reasonable request.
