# Supplementary material for: Mitochondrial Intercellular Transfer via Platelets After Physical Training Exerts Neuro‐Glial Protection Against Cerebral Ischemia
Source: MedComm (2020). 2026 Jan 15;7(2):e70590. doi: 10.1002/mco2.70590 (PMC12805465; doi:10.1002/mco2.70590)
Supplement: Supplementary file 1 — Supporting File 1: mco270590‐sup‐0001‐SuppMat.docx. [file MCO2-7-e70590-s004.docx]

**SUPPLEMENTAL MATERIAL**

**Mitochondrial intercellular transfer via platelets after physical training exerts neuro-glial protection against cerebral ischemia.**

Toshiki Inaba^1^, PhD; Nobukazu Miyamoto^1^, MD, PhD; Kenichiro Hira^1^, MD, PhD; Chikage Kijima^1^, MD, PhD; Yoshifumi Miyauchi^1^, MD; Hai-Bin Xu^1^; Yuji Ueno^1,2^, MD, PhD; Nobutaka Hattori^1,3^, MD, PhD

1) Department of Neurology, Juntendo University School of Medicine, Tokyo, Japan.

2) Department of Neurology, University of Yamanashi, Yamanashi, Japan

3) Neurodegenerative Disorders Collaborative Laboratory, RIKEN Center for Brain Science, Saitama, Japan

Corresponding author: Nobukazu Miyamoto, MD, PhD, and Nobutaka Hattori1,3, MD, PhD

Department of Neurology, Juntendo University School of Medicine, 2-1-1 Hongo, Bunkyo-ku, Tokyo 113-0033, Japan Telephone: +81-3-3813-3111 Fax: +81-3-5684-0476 E-mail:nobu-m@juntendo.ac.jp (Nobukazu Miyamoto), nhattori@juntendo.ac.jp (Nobutaka Hattori)

**Contents:**

- Supplement figure

- Supplement file legends

**Supplement figure**

**
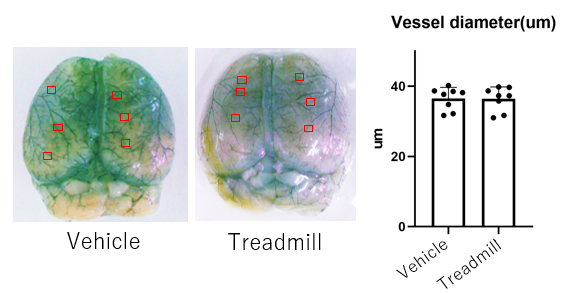
**

**Comparing vessel diameter at ACA-MCA anastomosis**

Vessel diameter was analyzed at 6 points of ACA-MCA anastomosis (each group n=8). After treadmill training, vessel diameters were no difference in vehicle and treadmill training group.

**Supplement files legends**

timelaps_OPCmito, timelaps_ASTmito, timelaps_neuronmito

Mitochondria labeled with FITC by mitoblight were administered to various cells (oligodendrocyte progenitor cells [OPCs]; timelaps_OPCmito, astrocytes; timelaps_ASTmito, neurons; timelaps_neuronmito) labeled with TRITC by mitoblight. Immediately after administration, the plate was allowed to stand for 1 hour because of the appearance of cloudiness in the plate, and images were taken every 10 minutes up to 6 hours after administration.

Mitochondria colored green can be observed changing to yellow as they are taken up over time by various cells in which the resident is a red mitochondrial cells.
